# Improving tremor response to focused ultrasound thalamotomy

**DOI:** 10.1093/braincomms/fcad165

**Published:** 2023-05-22

**Authors:** James M Holcomb, Rajiv Chopra, Fabricio S Feltrin, Mazen Elkurd, Rasheda El-Nazer, Lauren McKenzie, Padraig O’Suilleabhain, Joseph A Maldjian, William Dauer, Bhavya R Shah

**Affiliations:** Focused Ultrasound Lab and Program, Department of Radiology, UTSW Medical Center, Dallas, TX 75235, USA; Focused Ultrasound Lab and Program, Department of Radiology, UTSW Medical Center, Dallas, TX 75235, USA; Focused Ultrasound Lab and Program, Department of Radiology, UTSW Medical Center, Dallas, TX 75235, USA; Department of Neurology, UTSW Medical Center, Dallas, TX 75235, USA; Department of Neurology, UTSW Medical Center, Dallas, TX 75235, USA; Focused Ultrasound Lab and Program, Department of Radiology, UTSW Medical Center, Dallas, TX 75235, USA; Department of Neurology, UTSW Medical Center, Dallas, TX 75235, USA; Focused Ultrasound Lab and Program, Department of Radiology, UTSW Medical Center, Dallas, TX 75235, USA; Department of Neurology, UTSW Medical Center, Dallas, TX 75235, USA; O’Donnell Brain Institute, UTSW Medical Center, Dallas, TX 75235, USA; Focused Ultrasound Lab and Program, Department of Radiology, UTSW Medical Center, Dallas, TX 75235, USA; O’Donnell Brain Institute, UTSW Medical Center, Dallas, TX 75235, USA; Department of Neurological Surgery, UTSW Medical Center, Dallas, TX 75235, USA; Advanced Imaging Research Center, UTSW Medical Center, Dallas, TX 75235, USA; Center for Alzheimer’s and Neurodegenerative Diseases, UTSW Medical Center, Dallas, TX 75235, USA

**Keywords:** focused ultrasound, FUS, HIFU, essential tremor, diffusion tensor imaging

## Abstract

MRI-guided high-intensity focused ultrasound thalamotomy is an incisionless therapy for essential tremor. To reduce adverse effects, the field has migrated to treating at 2 mm above the anterior commissure-posterior commissure plane. We perform MRI-guided high-intensity focused ultrasound with an advanced imaging targeting technique, four-tract tractography. Four-tract tractography uses diffusion tensor imaging to identify the critical white matter targets for tremor control, the decussating and non-decussating dentatorubrothalamic tracts, while the corticospinal tract and medial lemniscus are identified to be avoided. In some patients, four-tract tractography identified a risk of damaging the medial lemniscus or corticospinal tract if treated at 2 mm superior to the anterior commissure-posterior commissure plane. In these patients, we chose to target 1.2–1.5 mm superior to the anterior commissure-posterior commissure plane. In these patients, post-operative imaging revealed that the focused ultrasound lesion extended into the posterior subthalamic area. This study sought to determine if patients with focused ultrasound lesions that extend into the posterior subthalamic area have a differnce in tremor improvement than those without. Twenty essential tremor patients underwent MRI-guided high-intensity focused ultrasound and were retrospectively classified into two groups. Group 1 included patients with an extension of the thalamic-focused ultrasound lesion into the posterior subthalamic area. Group 2 included patients without extension of the thalamic-focused ultrasound lesion into the posterior subthalamic area. For each patient, the percent change in postural tremor, kinetic tremor and Archimedes spiral scores were calculated between baseline and a 3-month follow-up. Two-tailed Wilcoxon rank-sum tests were used to compare the improvement in tremor scores, the total number of sonications, thermal dose to achieve initial tremor response, and skull density ratio between groups. Group 1 had significantly greater postural, kinetic, and Archimedes spiral score percent improvement than Group 2 (*P* values: 5.41 × 10^−5^, 4.87 × 10^−4^, and 5.41 × 10^−5^, respectively). Group 1 also required significantly fewer total sonications to control the tremor and a significantly lower thermal dose to achieve tremor response (*P* values: 6.60 × 10^−4^ and 1.08 × 10^−5^, respectively). No significant group differences in skull density ratio were observed (*P* = 1.0). We do not advocate directly targeting the posterior subthalamic area with MRI-guided high-intensity focused ultrasound because the shape of the focused ultrasound lesion can result in a high risk of adverse effects. However, when focused ultrasound lesions naturally extend from the thalamus into the posterior subthalamic area, they provide greater tremor control than those that only involve the thalamus.

## Teaser

Holcomb *et al.* demonstrates that when using four-tract tractography to target the decussating and non-decussating dentatorubrothalamic tracts with MRI-guided high-intensity focused ultrasound at 1.2–1.5 mm superior to the anterior commissure- posterior commissure plane, the focused ultrasound lesion extends into the posterior subthalamic area and further improves tremor control.

## Graphical Abstract

**Graphical abstract:**
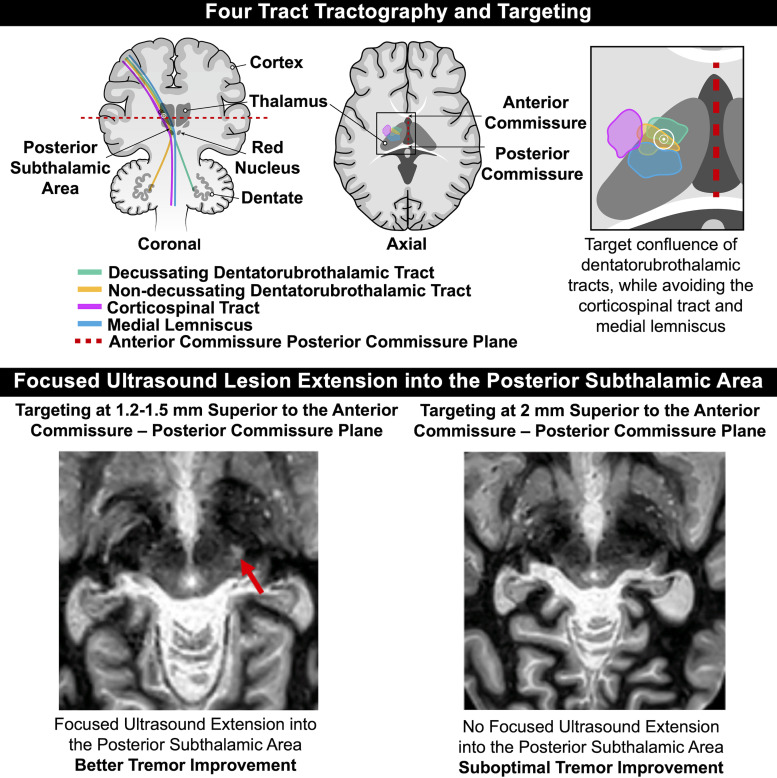

## Introduction

Magnetic resonance guided high intensity focused ultrasound (MRgHIFU) thalamotomy is a Federal Drug Administration-approved, incisionless, therapy for essential tremor (ET). Targeting using indirect coordinates has been a workhorse of neurosurgery and was developed because the MRgHIFU thalamotomy target, the ventral intermediate nucleus (VIM), cannot be seen on conventional high-resolution MRI.^1^ Targeting with indirect coordinates relies on measuring distances from key anatomic landmarks and suffers from a lack of precision due to human anatomic variation and variability in the indirect coordinates used among centres.^2^

In the landmark multi-centre clinical trial by Elias *et al*.,^3^ MRgHIFU targeting with indirect coordinates was performed at the level of the anterior–posterior commissural (AC–PC) plane. In this clinical trial, 36% of patients developed gait disturbances and 38% developed paresthaesia or numbness. These adverse effects (AEs) persisted at 12 months in 9% and 14% of patients, respectively. The AEs with MRgHIFU thalamotomy were partially thought to result from an extension of the focused ultrasound (FUS) lesion inferior to the AC–PC plane. As a result of these findings, the majority of centres advocate moving the indirect coordinates to 2.0 mm superior to the AC–PC plane thereby preventing inferior extension of the lesion. It is widely believed that by moving the FUS target 2.0 mm superior to the AC–PC plane, the AEs profile of MRgHIFU thalamotomy is improved. However, this was not supported by the largest retrospective analysis done on MRgHIFU for ET.^4^

Although many deep brain stimulation (DBS) centres also target the VIM, others advocate targeting the posterior subthalamic area (PSA) for optimal tremor control.^5,6^ The PSA is an anatomical term that includes several closely related structures including the caudal zona incerta, the decussating dentatorubrothalamic tract (dDRTT) and non-decussating dentatorubrothalamic tract (ndDRTT), pre-lemniscal radiations, and pallidothalamic white matter tracts. The PSA borders are posterior and medial to the internal capsule, lateral to the red nucleus, and anterior and medial to the medial lemniscus. Due to the large heterogeneous and complex anatomy of the PSA, there is variability in how it is defined.^7–9^ As the dDRTT and ndDRTT are principal components of the PSA,^10–12^ the PSA was defined as the posterior confluence of the dDRTT and ndDRTT at the level of the midbrain in this study.

Our group has developed a novel targeting method, four-tract tractography, that incorporates decades of knowledge provided by anatomical studies, indirect targeting, advanced structural imaging methods, MR diffusion tractography and functional tremor response.^13–24^ Four-tract tractography, a precision medicine approach, refines the indirect target based on imaging surrogates of key white matter tracts for tremor control using patient-specific treatment coordinates. The critical white matter targets for tremor control are the dDRTT and ndDRTT, while the medial lemniscus (ML) and corticospinal tracts (CST) are identified to be avoided. The overlap of the MRgHIFU lesion with the dDRTT and ndDRTT has been shown to correlate with improved tremor response.^23^ Therefore, in our practice we routinely target the posterior confluence of these two white matter tracts for MRgHIFU thalamotomy.^13^ Four-tract tractography decreases treatment time, reduces AEs, decreases the thermal dose required to see an initial tremor response, and maintains extremity and axial tremor response from 3 months to 1 year.^13,25^

Although we initially accepted the standard of treating 2.0 mm superior to the AC–PC plane, in some patients four-tract tractography further informed the targeting decision, and we chose to treat at 1.2–1.5 mm superior to the AC–PC plane to avoid damaging the ML and CST. In those patients treated at 1.2–1.5 mm superior to the AC–PC plane, the MRgHIFU lesion extended into the PSA. This manuscript further refines the four-tract tractography method of MRgHIFU thalamotomy for the treatment of ET. It provides our clinical experience comparing 20 patients who either had FUS lesion extension into the PSA (*n* = 10) or did not have FUS lesion extension into the PSA (*n* = 10) and offers a mechanistic explanation for differences in tremor outcomes.

## Materials and methods

### Patient selection

**Figure 1:**
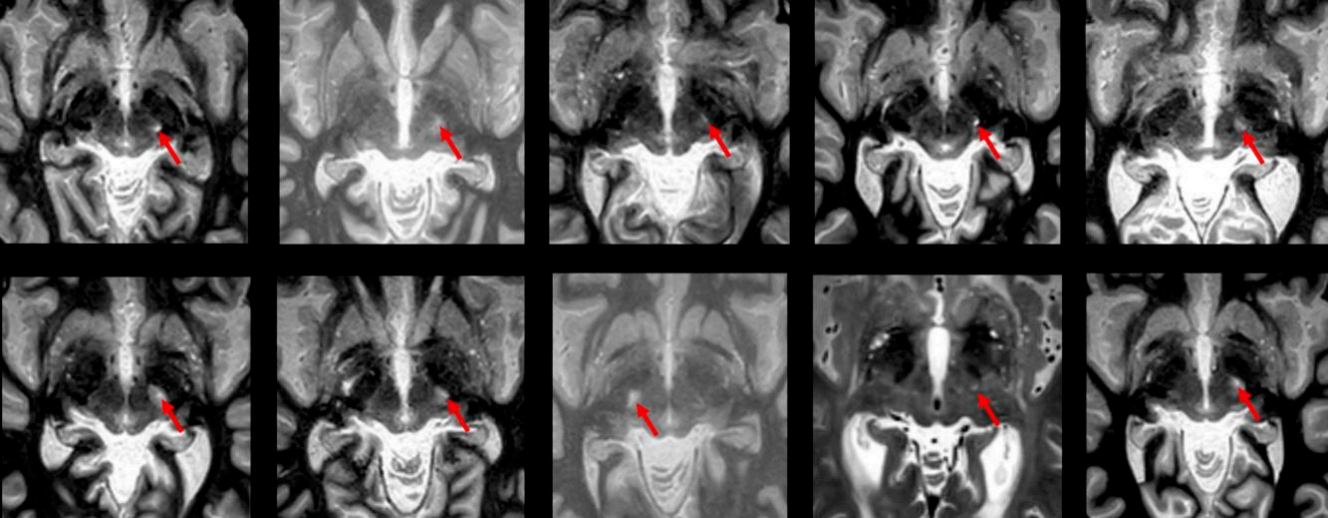
**Axial T2W and FGATIR images from the 3-month post-operative MRI demonstrate the FUS lesion extension into the PSA in all ten patients targeted at 1.2–1.5 mm superior to the AC–PC plane (Group 1).** In each image, the FUS lesion appears as a hyperintense spheroid located lateral to the red nucleus and posteromedial to the subthalamic nucleus. The FUS lesion in each image is indicated by a red arrow.

**Figure 2:**
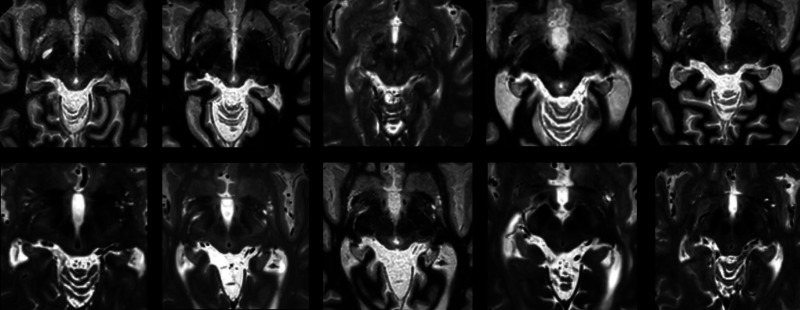
Axial T2W and FGATIR images from the 3-month post-operative MRI demonstrate no extension of the FUS lesion into the PSA in all ten patients targeted at 2.0 mm superior to the AC–PC plane (Group 2).

The data presented in this manuscript reflect 20 ET patients who underwent MRgHIFU from April 2021 to May 2022. Patients were required to have a baseline, a 3-month follow-up, and an AE assessment by a movement disorders neurologist with the ET rating assessment scale (TETRAS) tremor score assigned. The definition of ET followed the consensus statement on the classification of tremors from the Task Force on Tremor of the International Parkinson and Movement Disorder Society.^26^ Patients with ‘mixed tremor’, ET+ and tremor-dominant Parkinson’s disease were excluded from this study. Using these criteria, 10 ET patients who were targeted 1.2–1.5 mm superior to the AC–PC plane with FUS lesion extension into the PSA (Group 1) were first identified (Fig. 1). Subsequently, an equal number of ET patients (*n* = 10) with targeting at the standard 2.0 mm superior to AC–PC without FUS lesion extension into the PSA (Group 2) were also identified (Fig. 2).

### Clinical assessment

A movement disorders neurologist is part of our treatment team and performs neurologic assessments before, during, and after the treatment. Assessments include a cranial nerve exam, motor strength, coordination evaluation, gait testing, superficial and deep sensitivity in all extremities and face, tremor assessment with finger-to-nose testing, hands positioned at near and far, spirals and axial tremor assessment including voice tremor. The gait assessment evaluates ataxia with tandem walking and stance in stationary and tandem positions. Truncal ataxia is further assessed by having patients sit with arms outstretched and eyes closed without foot support. AEs related to the procedure were recorded at each follow-up appointment.

### The essential tremor rating assessment scale (TETRAS) assessment

Three movement disorders neurologists independently rated postural tremor and kinetic tremor using the TETRAS scale.^27^ When making their assessments, each neurologist was blinded to the targeting location and whether the FUS lesion extended into the PSA. To simplify quantification and analysis, the postural tremor score that was higher (either upper limbs held forward and horizontally or with upper limbs extended laterally and horizontally with the elbows flexed and hands positioned close to each other near the chin) was used to monitor postural tremor before and after treatment. The kinetic tremor was scored using finger-nose-finger movements.

### Spiral assessment

Three movement disorders neurologists and one movement disorder trained physician assistant independently and blindly rated Archimedes spirals using the modified Washington Heights-Inwood Genetic Study of Essential Tremor (WHIGET) Rating Scale^28^ at baseline and three months after MRgHIFU. The physician assistant was trained by our movement disorders neurologists to independently evaluate tremor in clinic, with 8 years of experience in both clinical and research environments. The agreement between raters was quantified with a kappa value of 0.95. A blinded consensus conference was held to finalize scores. All raters were blinded to the targeting location and whether the FUS lesion extended into the PSA.

### MRI acquisition

For all patients, a 60-minute MRI scan with diffusion tensor imaging (DTI) was performed on a Phillips 3 T MR Scanner (Philips, Best, The Netherlands). The sequences includes isotropic T2-weighted (T2W) three-dimensional turbo field echo [field of view (FOV) 24 cm, matrix 268 × 268 mm, repetition time (TR) = 2500, echo time (TE) = 255.56, thickness 0.9 mm, gap = 0 mm, spacing = 0.9 mm], fast grey matter acquisition T1 inversion recovery (FGATIR) (FOV 25 cm, matrix 256 × 256, TR = 6.615, TE = 2.949, thickness = 0.9 mm, gap = 0, spacing = 0.9 mm), axial three-dimensional T1 turbo field echo (FOV = 24 cm, matrix 268 × 187, TR = 8588 ms, TE = 3.93 ms, gap = 0, spacing = 0.9 mm) and 32 direction DTI (FOV 24 × 24 × 15 cm, matrix 96 × 96, B value = 800, TR = 3400, TE = 84.5, acquisition voxel = 2.5 mm, thickness 2.5 mm, spacing 2.5 mm, gap = 0, SNR = 0.99, slices = 60 and Halfscan factor = 0.84).

### DTI registration with structural images and distortion correction

The images are uploaded to BrainLab (Munich, Germany). The DTI images are rigidly co-registered to both structural FGATIR and three-dimensional T2W sequences using Siemens Image Fusion, both with a 0.7 mm isotropic resolution. Co-registration and distortion correction of DTI and anatomical images is based on inverse contrast normalization which is performed in Brainlab elements.^29^ A corrected DTI image set is generated and utilized for fibre tracking.

### Treatment

We follow a standard patient preparation protocol that has been previously described.^13^ The burned-in image is first registered to the head CT. Fiducials are placed at target locations as determined during treatment planning. ML and CST safety margins are verified. A real-time sagittal constructive interference in steady state sequence (FOV 32 cm, matrix 320 × 240 mm, TR = 8.64, TE = 4.32 thickness 1.5 mm, gap = 0.68 mm) is obtained. The AC and PC are identified, and the images are reformatted in three planes. The midline is marked on a coronal image. The real-time MRI is registered to the treatment plan. After each treatment sonication, the patient is evaluated for tremor response and side effects. Our desired thermal dose at each target is 56–57 °C. The temperature did not exceed 45–47 °C during alignment sonications. Although we initially accepted the standard of treating 2.0 mm superior to the AC–PC plane, in some patients, four-tract tractography revealed that the CST or the ML were susceptible to damage at 2.0 mm superior to the AC–PC plane. In these patients, the target was moved to 1.2–1.5 mm superior to the AC–PC plane.

### Posterior subthalamic area evaluation

The red nucleus and subthalamic nucleus are routinely used as landmarks on T2W images to identify the PSA.^30,31^ Accordingly, one board-certified neuroradiologist inspected both T2W and FGATIR images acquired three months post-procedure to determine whether the lesion extended into the PSA.

### Statistical analysis

Each patient’s percent tremor improvement was calculated using the TETRAS postural, kinetic, and Archimedes spiral assessments. For each of the tremor assessments, a two-tailed Wilcoxon rank-sum test was used to compare the percent improvement in ET score between the patients with lesion extension into the PSA and those without lesion extension into the PSA. Additionally, two-tailed Wilcoxon rank-sum tests were performed to evaluate differences in skull density ratio, thermal dose at first tremor response, and total number of sonications between groups. Statistical analyses were performed with alphas of 0.05 using the coin package^32^ in R 4.2.3 [R Core Team (2023), Vienna, Austria].

## Results

### Patients

A total of twelve male and eight female patients (age range 56–87 years old, mean age 74.5 years old, standard deviation 8.41 years) were included in the study. Patients in Group 1, in which the FUS lesion extended into the PSA, were on average, 74.3 years old, standard deviation of 8.2 years. Patients in Group 2, in which the FUS lesion did not extend into the PSA, were on average, 74.7 years old, standard deviation of 9.0 years. Each group contained an equal number of male (six) and female (four) patients. The average skull density ratio was 0.572 (standard deviation of 0.090) for Group 1 and 0.578 (standard deviation of 0.0820) for Group 2. At least two FUS lesions were created in all patients. In eight patients, to address residual axial or voice tremor, a third FUS lesion was created. Each group contained an equal number of patients (four) with a third lesion site. In the study cohort, six patients reported transient imbalance (30%) that subsided after 3 weeks, and one patient reported numbness (5%) that subsided after 1 week. Group 1 had fewer AEs (two) than Group 2 (five). Dates for the procedure were intermixed and did not impact patient stratification. The terminal target treatment temperature was 57 °C for both groups.

### Statistics tests

**Figure 3:**
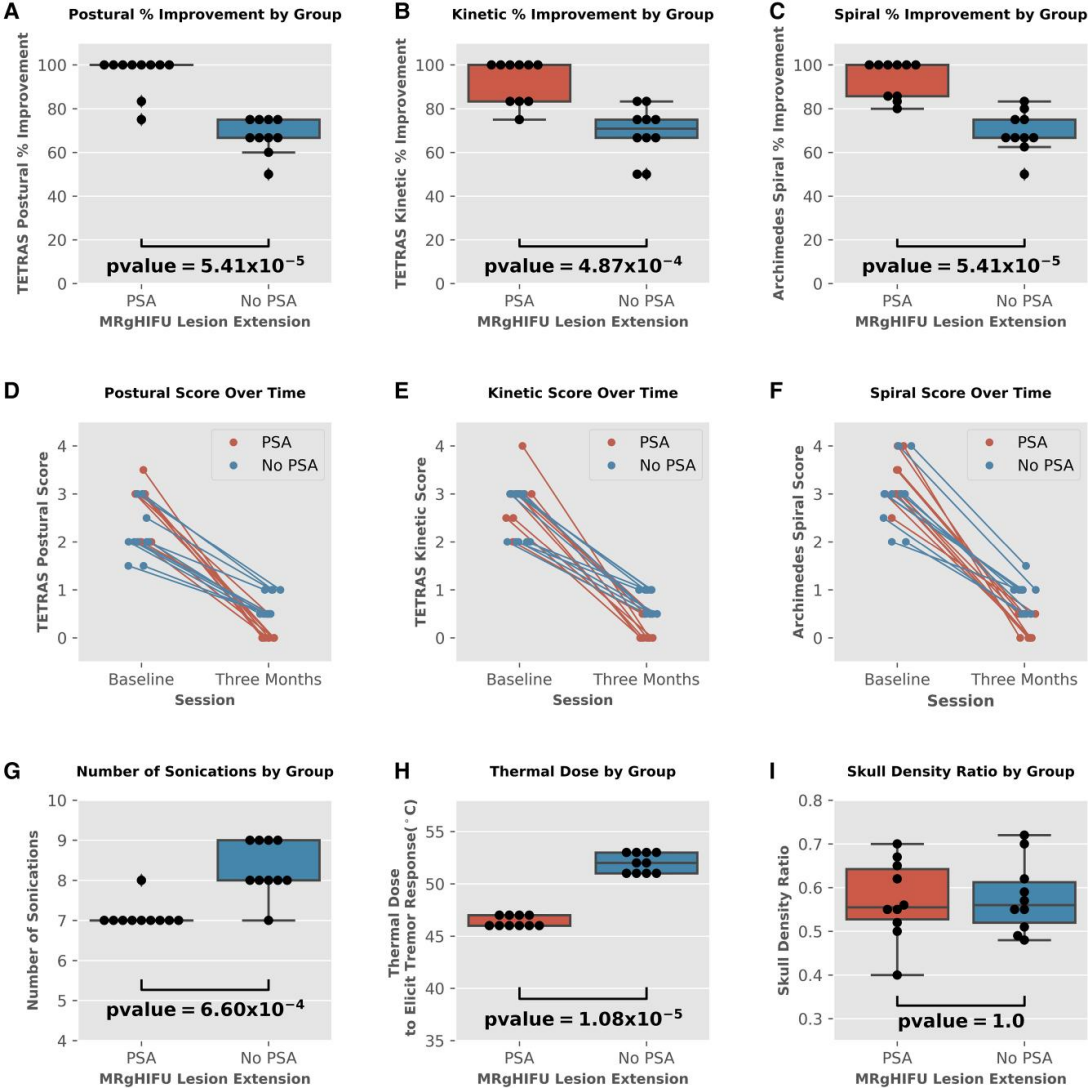
**Results of all analyses between groups.** (**A–C**) Percent tremor improvement comparisons for all tremor assessments. Orange boxplots on the left and blue boxplots on the right of each graph, respectively display the distribution of percent ET improvement for the patients with FUS lesions that extended into the PSA and lesions that did not extend into the PSA. Black dots indicate percent improvement in ET score for each patient. (**A**) Percent improvement in postural TETRAS scores by group. (**B**) Percent improvement in kinetic TETRAS scores by group. (**C**) Percent improvement in TETRAS Archimedes spiral scores by group. (**D–F**) ET scores for each patient at baseline and 3-month follow-up. Orange dots indicate scores for patients with FUS lesion extension into the PSA. Blue dots indicate scores for patients with FUS lesions that did not extend into the PSA. Diagonal lines connect the baseline and follow-up scores for each patient. (**D**) Postural TETRAS assessment scores at baseline and 3-month follow-up for those with FUS lesions extending into the PSA and those without lesion extension into the PSA. (**E**) Kinetic TETRAS assessment scores at baseline and 3-month follow-up for those with FUS lesions extending into the PSA and those without lesion extension into the PSA. (**F**) TETRAS Archimedes spiral assessment scores at baseline and 3-month follow-up for those with FUS lesions extending into the PSA and those without lesion extension into the PSA. (**G–I**) FUS parameter comparisons. (**G**) Number of sonications by group. (**H**) Thermal dose to elicit tremor response by group. (**I**) Skull density ratio by group. The *P* values for each Wilcoxon rank-sum test (**A–C**) and (**G–I**) are displayed below each boxplot distribution. For all tests, *n* = 10 in each group.

Wilcoxon rank-sum tests for all three neurological assessments demonstrated a significantly greater percent improvement in tremor score in patients with FUS lesion extension into the PSA than those without FUS lesion extension into the PSA. Wilcoxon rank-sum test *P* values for TETRAS postural, kinetic, and Archimedes spiral assessments were 5.41 × 10^−5^, 4.87 × 10^−4^, and 5.41 × 10^−5^, respectively. Average TETRAS postural, kinetic, and Archimedes spiral assessment percent improvement for Group 1 were 95.8%, 92.5% and 93.5%, respectively. In comparison, average TETRAS postural, kinetic, and spiral assessment percent improvement for Group 2 were 67.7%, 69.2% and 69.3%, respectively. Group 1 also required a significantly lower total number of sonications during the procedure (*P* = 6.60 × 10^−4^) and required a significantly lower thermal dose to elicit an initial tremor response (*P* = 1.08 × 10^−5^) than Group 2. There was no significant difference in skull density ratio between groups (*P* = 1.0). Group 1 required 7.1 average total sonications, while Group 2 required 8.3 average total sonications to achieve satisfactory tremor control. The average thermal dose to elicit tremor response for Group 1 was 46.4 °C, and 52.0 °C for Group 2. The average skull density ratio for Group 1 was 0.572, while the average skull density for Group 2 was 0.578. Demographic information and MRgHIFU statistics for each subject are provided in Table 1. Visualization of all group comparisons is found in Fig. 3.

**Table 1:** Patient demographics, procedural parameters and results

Subject ID	Number of alignment sonications	Total number of sonications	Thermal dose at tremor response (°C)	Skull density ratio	Number of lesions	TETRAS postural tremor baseline score	TETRAS postural tremor follow-up score	TETRAS kinetic tremor baseline score	TETRAS kinetic tremor follow-up score	TETRAS Archimedes spiral baseline score	TETRAS Archimedes spiral follow-up score	AEs
HIFU_001^1^	3	7	47	0.55	2	3.0	0.0	3.0	0.0	3.0	0.0	None
HIFU_002^1^	3	7	46	0.52	2	2.0	0.5	2.0	0.5	2.5	0.5	None
HIFU_003^1^	3	7	47	0.62	3	3.5	0.0	3.0	0.0	3.0	0.0	None
HIFU_004^1^	3	7	46	0.67	3	3.0	0.0	4.0	0.0	4.0	0.0	None
HIFU_005^1^	3	7	47	0.50	2	2.0	0.0	2.5	0.0	3.0	0.0	None
HIFU_006^1^	3	7	46	0.65	2	3.0	0.0	3.0	0.0	4.0	0.0	Imbalance ∼3 weeks
HIFU_007^1^	4	7	47	0.40	3	2.0	0.0	2.5	0.0	3.0	0.0	None
HIFU_008^1^	3	7	46	0.70	3	3.0	0.5	3.0	0.5	3.5	0.5	None
HIFU_009^1^	3	8	46	0.55	2	3.0	0.0	3.0	0.5	3.5	0.5	None
HIFU_010^1^	3	7	46	0.56	2	3.0	0.0	3.0	0.5	3.0	0.5	Imbalance ∼2 weeks
HIFU_011^2^	3	9	51	0.57	2	2.0	0.5	2.0	0.5	2.5	0.5	Imbalance ∼3 weeks
HIFU_012^2^	3	8	52	0.55	2	2.0	0.5	2.0	1.0	3.0	1.0	Imbalance ∼3 weeks
HIFU_013^2^	3	9	53	0.49	2	1.5	0.5	3.0	0.5	4.0	1.0	None
HIFU_014^2^	3	8	53	0.72	2	3.0	1.0	3.0	1.0	4.0	1.5	None
HIFU_015^2^	3	8	53	0.51	3	2.0	0.5	3.0	1.0	3.0	1.0	None
HIFU_016^2^	4	8	51	0.48	3	1.5	0.5	2.0	0.5	2.0	1.0	None
HIFU_017^2^	3	9	52	0.62	3	2.0	0.5	3.0	0.5	3.0	1.0	None
HIFU_018^2^	3	8	51	0.55	2	2.5	1.0	2.0	0.5	2.0	0.5	Imbalance ∼1 week
HIFU_019^2^	4	7	53	0.70	2	2.0	1.0	3.0	1.0	3.0	0.5	Imbalance ∼3 weeks
HIFU_020^2^	3	9	51	0.59	3	3.0	1.0	2.0	1.0	3.0	1.0	Numbness ∼1 week

Subject IDs with superscripted 1 had FUS targets placed 1.2–1.5 mm above AC–PC and the resultant FUS lesion extended into the PSA (Group 1), while subject IDs with superscripted 2 had FUS targets placed 2.0 mm above AC–PC and the resultant FUS lesion did not extend into the PSA (Group 2).

## Discussion

In this manuscript, we demonstrate a statistically significant improvement in postural and kinetic tremor as well as Archimedes spiral scores when using four-tract tractography to target the dDRTT and ndDRTT at 1.2–1.5 mm (FUS lesion extension into the PSA) instead of 2.0 mm (no FUS lesion extension into the PSA) superior to the AC–PC plane. This improvement is seen in the absence of an increased incidence of AEs. We also demonstrate that when targeting at 1.2–1.5 mm superior to the AC–PC plane, fewer number of total sonications are required to achieve satisfactory tremor control and a lower temperature is required to elicit tremor response.

The conventional target for treating ET is the VIM nucleus of the thalamus. However, the VIM cannot be clearly delineated on standard, high-resolution MRI. Therefore, indirect targeting methods are commonly employed. Among centres, the VIM indirect coordinates are variably defined at 13–15 mm lateral to the mid-commissural line or 11–11.5 mm lateral from the wall of the third ventricle; a quarter of the AC–PC distance anterior to the PC at the level of the AC–PC plane or 6 mm anterior to the PC, and at the level of the AC–PC plane.^2,9^ Unlike other ablative technologies or DBS, the MRgHIFU lesion is a prolate spheroid with its long-axis extending vertically from the supero-medial to infero-lateral direction. Extension of the HIFU lesion below the AC–PC plane was thought to account for the high number of AEs encountered in initial clinical studies.^3^ Accordingly, MRgHIFU centres have modified the indirect coordinates to treat 2.0 mm superior to the AC–PC plane. However, in the largest recent retrospective MRgHIFU study, modifying the indirect coordinates to 2 mm superior to the AC–PC plane did not significantly reduce the AEs profile of MRgHIFU thalamotomies.^4^ In agreement with these findings, we also found no increase in AEs when targeting 1.2–1.5 mm superior to the AC–PC plane. Instead, we observed better tremor improvement and fewer AEs in those targeted at 1.2–1.5 mm superior to the AC–PC than those treated at 2.0 mm superior to the AC–PC plane.

**Figure 4:**
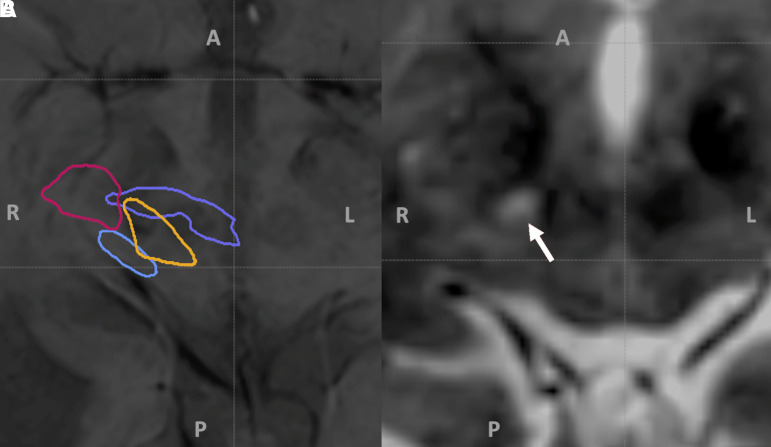
**Axial post-operative susceptibility-weighted and T2W images displaying lesions in the right PSA.** (**A**) Corticospinal tract (magenta), medial lemniscus (blue), non-decussating dentatorubrothalamic tract (gold), and decussating dentatorubrothalamic tract (purple) overlayed on susceptibility-weighted image at the level of the PSA. Hypointensity in centre of tracts identifies the FUS lesion as it extends along the decussating and non-decussating dentatorubrothalamic tracts and avoids extension into the corticospinal and medial lemniscus tracts. (**B**) Hyperintensity indicated by the white arrow on the T2W image identifies the lesion site at the level of the right PSA. A, P, R and L in each image indicate radiological anterior, posterior, right and left, respectively.

When we target the posterior confluence of the dDRTT and ndDRTT 1.2–1.5 mm superior to the AC–PC plane, the lesion can be identified extending into the PSA on post-procedural imaging. When we define the PSA as the posterior confluence of the dDRTT and ndDRTT at the level of the midbrain and overlay the structural images with four-tract tractography, we discover that the PSA is the only treatable site other than the thalamus that has maximum overlap on both the dDRTT and ndDRTT (Fig. 4). A recent study evaluating the efficacy of DBS for ET revealed that ‘sweet spots’ reported by multiple groups all share overlap with a common tract, the DRTT.^33^ DTI studies have also demonstrated that the dentatorubrothalamic tracts along with pallidothalamic fibres are the principal components of the PSA.^10–12^ Accordingly, we believe that by using four-tract tractography instead of indirect coordinates to target the dDRTT and ndDRTT at 1.2–1.5 mm superior to the AC–PC plane, there is a corresponding extension of the thalamic lesion inferiorly into the portion of the PSA that contributes to the improved tremor response. Additionally, this is consistent with long-standing belief that sub-thalamotomy is important for tremor control. Although the rationale for doing so has remained unclear until now.

For over two decades, the Oxford neurosurgery group has employed an alternative targeting methodology for treating ET. Their practice is to target the border of the VIM, the ventralis oralis posterior, and the PSA. However, in a paper employing this technique, Jameel *et al*. report that 8 out of 14 patients (57%) had AEs, including dysarthria (*n* = 1), unsteady gait (*n* = 4) or chorea (*n* = 2) at 3 months post-procedure.^9^ These AEs decreased to chorea (*n* = 1) and unsteady gait (*n* = 1) at 24 months. The high incidence of AEs reported by the Oxford group can be explained by the difference in a HIFU lesion when compared to a DBS lead, in which placement in the PSA is well-tolerated.^34–36^ As the HIFU lesion is a prolate spheroid, directly targeting the PSA is more likely to damage adjacent structures. In contrast, because we do not directly target the PSA, but instead use the natural shape of the HIFU lesion and allow it to extend into the PSA, we do not see an increased incidence of AEs. In addition, identifying the CST and ML through four-tract tractography allows us to reduce our AE profile.^13,37–39^ Further, the benefit of the second PSA lesion by the Oxford group was unclear because they created the PSA lesion after a thalamic lesion.^9^ This is in contrast to our approach in which the first thalamic lesion extends into the PSA and provides immediate tremor control.

There are several limitations to this manuscript. First, we report patient outcomes at 3 months. Although this is an established time point to gauge response to therapy as oedema has resolved, prior studies suggest that there is some tremor recurrence at 12 months. While other groups have reported an improvement in AEs at 12 months, the only AEs we encountered were transient subjective imbalance and numbness, which resolved less than 1 month after treatment. Second, this is a retrospective study design. However, this report provides a refined technique to improve tremor control with MRgHIFU thalamotomy. Our experience suggests that this is a reliable and precise method to achieve superior tremor control. Further multicenter prospective validation and formal comparative evaluation are warranted to further assess the reproducibility, clinical effectiveness, and safety of this approach because preliminary open-label analysis suggests promise.

## Conclusion

While we do not advocate targeting the PSA directly, this study demonstrates that the natural extension of the MRgHIFU lesion into the PSA provides greater tremor control than thalamic lesions alone. When using four-tract tractography to target the posterior confluence of the dDRTT and ndDRTT at 1.2–1.5 mm rather than 2.0 mm superior to the AC–PC plane, we observe improved tremor control, fewer total required sonications, and a lower thermal dose required to elicit tremor control response. We further use anatomic and technical knowledge to offer mechanistic insight into tremor improvement when using four-tract tractography.^40,41^

## Data Availability

The data that support the findings of this report are available on request from the corresponding author. The data are not publicly available due to information that could compromise the privacy of patients.

## Abbreviations

AC =: anterior commissure
AEs =: adverse effects
CST =: corticospinal tract
DBS =: deep brain stimulation
dDRTT =: decussating dentatorubrothalamic tract
DTI =: diffusion tensor imaging
ET =: essential tremor
FGATIR =: fast grey matter acquisition T1 inversion recovery
FOV =: field of view
FUS =: focused ultrasound
ML =: medial lemniscus
MRgHIFU =: magnetic resonance guided high-intensity focused ultrasound
ndDRTT =: non-decussating dentatorubrothalamic tract
PC =: posterior commissure
PSA =: posterior subthalamic area
T2W =: T2-weighted
TE =: echo time
TETRAS =: the essential tremor rating assessment scale
TR =: repetition time
VIM =: ventral intermediate nucleus
